# Supplementary material for: High and asymmetric somato-dendritic coupling of V1 layer 5 neurons independent of visual stimulation and locomotion
Source: eLife. 2019 Dec 27;8:e49145. doi: 10.7554/eLife.49145 (PMC6974354; doi:10.7554/eLife.49145)
Supplement: Transparent reporting form [file elife-49145-transrepform.docx]

***eLife’s* transparent reporting form**

We encourage authors to provide detailed information *within their submission* to facilitate the interpretation and replication of experiments. Authors can upload supporting documentation to indicate the use of appropriate reporting guidelines for health-related research (see [EQUATOR Network](http://www.equator-network.org/%20)), life science research (see the [BioSharing Information Resource](https://biosharing.org/)), or the [ARRIVE guidelines](http://www.plosbiology.org/article/info:doi/10.1371/journal.pbio.1000412) for reporting work involving animal research. Where applicable, authors should refer to any relevant reporting standards documents in this form.

If you have any questions, please consult our Journal Policies and/or contact us: [editorial@elifesciences.org](mailto:editorial@elifesciences.org).

**Sample-size estimation**

- You should state whether an appropriate sample size was computed when the study was being designed
- You should state the statistical method of sample size computation and any required assumptions
- If no explicit power analysis was used, you should describe how you decided what sample (replicate) size (number) to use

Please outline where this information can be found within the submission (e.g., sections or figure legends), or explain why this information doesn’t apply to your submission:

Previous published data produced conflicting results regarding the correlation of calcium signals between sibling branches of layer 5 pyramidal neurons (Xu *et al*. 2012, Cichon and Gan 2015) and no such dataset was recorded in the primary visual cortex.

Similarly, no previous data have been published quantifying the distance-dependent attenuation of calcium signals in layer 5 pyramidal neurons in awake behaving animals.

Therefore, group variances could not be estimated in advance.

**Replicates**

- You should report how often each experiment was performed
- You should include a definition of biological versus technical replication
- The data obtained should be provided and sufficient information should be provided to indicate the number of independent biological and/or technical replicates
- If you encountered any outliers, you should describe how these were handled
- Criteria for exclusion/inclusion of data should be clearly stated
- High-throughput sequence data should be uploaded before submission, with a private link for reviewers provided (these are available from both GEO and ArrayExpress)

Please outline where this information can be found within the submission (e.g., sections or figure legends), or explain why this information doesn’t apply to your submission:

*Biological replicates*

Biological replicates in this study were individual neurons. For each neuron, a maximum of two pairs of compartments were recorded. The number of biological replicates is included in the main text and in each figure legend.

*Technical replicates*

Technical replicates for this study were individual trials which consisted of 65 seconds each in duration resulting in a change in fluorescence over time (∆F/F) for each neuron in the field of view. For each field-of-view, we acquired 8-12 trials in darkness and 12-20 trials during visual stimulation. The number of technical replicates is included in the Materials and methods section (Visual stimulation).

No outliers were removed in any of the datasets.

Criteria for designation of behavioural state conditions (locomotion versus stationary periods) is clearly described in the experimental procedures.

As described in the manuscript (discussion and Material and Methods), we systematically reconstructed our imaged neurons and excluded regions of interest where overlapping dendrites from other neurons were observed. We used the activity-independent marker tdTomato to correct for motion artefacts and excluded the recordings in which tdTomato marker was not detected.

**Statistical reporting**

- Statistical analysis methods should be described and justified
- Raw data should be presented in figures whenever informative to do so (typically when N per group is less than 10)
- For each experiment, you should identify the statistical tests used, exact values of N, definitions of center, methods of multiple test correction, and dispersion and precision measures (e.g., mean, median, SD, SEM, confidence intervals; and, for the major substantive results, a measure of effect size (e.g., Pearson's r, Cohen's d)
- Report exact p-values wherever possible alongside the summary statistics and 95% confidence intervals. These should be reported for all key questions and not only when the p-value is less than 0.05.

Please outline where this information can be found within the submission (e.g., sections or figure legends), or explain why this information doesn’t apply to your submission:

Statistical analyses are described in the main text, figure legends and Methods.

In short, we used:

- paired t-test to compare two groups when the same biological replicate was recorded in two different conditions.

- one-way ANOVA when we had to compare two or more independent (unrelated) groups.

- two-way ANOVA when we had to compare the mean differences between groups on two independent and interacting factors (e.g. visual stimulation and locomotion).

- three-way ANOVA when we had to compare the mean differences between groups on three independent and interacting factors (e.g. visual stimulation, locomotion, and event amplitude).

Exact p values are reported both in text and in the relevant figure legends.

For each key experimental result, we have described the summary statistics, n values, p-values and statistical test used.

Raw data (changes of fluorescence over time) are provided in all the main figures (figure 1-4) and in two supplementary figures.

Additionally, we provide two data source videos (supplementary video 1 and supplementary video 2).

(For large datasets, or papers with a very large number of statistical tests, you may upload a single table file with tests, Ns, etc., with reference to sections in the manuscript.)

**Group allocation**

- Indicate how samples were allocated into experimental groups (in the case of clinical studies, please specify allocation to treatment method); if randomization was used, please also state if restricted randomization was applied
- Indicate if masking was used during group allocation, data collection and/or data analysis

Please outline where this information can be found within the submission (e.g., sections or figure legends), or explain why this information doesn’t apply to your submission:

Criteria for designation of sensory and behavioural state conditions (Visual stimulation versus darkness and locomotion versus stationary periods) are clearly described in the experimental procedures.

**Additional data files (“source data”)**

- We encourage you to upload relevant additional data files, such as numerical data that are represented as a graph in a figure, or as a summary table
- Where provided, these should be in the most useful format, and they can be uploaded as “Source data” files linked to a main figure or table
- Include model definition files including the full list of parameters used
- Include code used for data analysis (e.g., R, MatLab)
- Avoid stating that data files are “available upon request”

Please indicate the figures or tables for which source data files have been provided:

Source data are provided for supplementary video 1 and supplementary video 2.
